# Longitudinal genomic prediction of cacao disease resilience identifies robust witches’ broom disease targets

**DOI:** 10.3389/fpls.2026.1837102

**Published:** 2026-06-15

**Authors:** Insuck Baek, Rakesh K. Upadhyay, Moon S. Kim, Lyndel W. Meinhardt, Ezekiel Ahn

**Affiliations:** 1 Environmental Microbial and Food Safety Laboratory, Agricultural Research Service, United States Department of Agriculture, Beltsville, MD, United States; 2 Department of Natural Sciences, College of Arts and Sciences, Bowie State University, Bowie, MD, United States; 3 Sustainable Perennial Crops Laboratory, Agricultural Research Service, United States Department of Agriculture, Beltsville, MD, United States

**Keywords:** breeding prioritization, cacao, disease resilience, genomic prediction, longitudinal phenotypes, relatedness-blocked cross-validation, repeated harvest, witches’ broom disease

## Abstract

**Introduction:**

Genomic studies in cacao (*Theobroma cacao* L.) have largely emphasized single-locus discovery, but less is known about whether repeated-harvest disease trajectories can be predicted from genome-wide markers to support long-term breeding objectives.

**Methods:**

We analyzed repeated-harvest phenotypes from 102 cacao accessions to derive longitudinal targets describing baseline performance and temporal change for healthy pod rate, frosty pod rot (FPR), and witches’ broom disease (WBD). Genomic prediction was evaluated using random and relatedness-blocked cross-validation with single nucleotide polymorphism (SNP) marker sets aligned to the Criollo and Matina reference genomes. Robustness was assessed across trajectory model specifications, cross-validation block structures, and multi-trait selection indices.

**Results:**

Mixed-model analyses demonstrated temporal repeatability ranging from 0.78 to 0.89 across four harvests, with particularly high accession-level consistency for WBD traits. Genomic prediction models showed that WBD-derived targets, particularly branch and flower-cushion WBD symptom components, yielded more consistent predictive performance than FPR or healthy pod rate. These signals remained supported after 10,000-label permutation testing, Benjamini–Hochberg false-discovery-rate correction, and multiple robustness analyses. Cross-reference comparisons indicated that WBD predictability was robust to the choice of reference genome, whereas other traits exhibited higher reference sensitivity. Finally, Pareto-based multi-trait prioritization identified a stable shortlist of candidate accessions optimizing both disease resilience and productivity.

**Discussion:**

Repeated-harvest disease phenotypes harbored reproducible accession-level temporal structure, with WBD-related longitudinal traits remaining genomically predictable across diverse validation settings. These findings indicate that repeated-harvest trajectory targets provide useful genomic-prediction endpoints for prioritizing resilient germplasm in perennial cacao breeding programs, while emphasizing the need for validation in larger multi-environment trials.

## 1 Introduction

Cacao (*Theobroma cacao* L.) is an economically significant tropical crop. Production is severely constrained by fungal diseases, notably frosty pod rot (FPR) and witches’ broom disease (WBD), caused by *Moniliophthora* species. Development of resistant cultivars represents the most viable strategy for long-term disease management (Wickramasuriya and Dunwell, 2018; Bekele and Phillips-Mora, 2019; Osorio-Guarín et al., 2020).

Genetic improvement in perennial crops is inherently slow due to extended juvenile phases and the requirement for long-term field evaluations. Genomic prediction (GP) addresses these limitations by using genome-wide markers to estimate breeding values, thereby enabling selection prior to phenotypic expression and reducing breeding cycle time (Isik, 2014; Cros et al., 2015; Seyum et al., 2022; Robinson et al., 2025). In cacao, GP has demonstrated utility for complex traits, including yield and disease resistance, by modeling genome-wide marker effects rather than relying solely on individual marker–trait associations (Romero Navarro et al., 2017; McElroy et al., 2018; Bekele et al., 2022). Previous studies using the Agrosavia diversity panel highlighted the polygenic nature of resistance and demonstrated the sensitivity of locus discovery to reference-genome choice (Osorio-Guarín et al., 2020; Ahn et al., 2025).

While GP is increasingly applied in perennial breeding, phenotypic targets are typically defined as static, aggregated values recorded at a single time point. However, traits such as disease severity and pod production are continuous processes shaped by repeated cycles of plant growth and environmental interactions. Longitudinal models that incorporate time-series phenotypic data have been shown to capture the temporal dynamics of trait expression, consequently improving genomic prediction accuracies in various crop species (Campbell et al., 2018; Baba et al., 2020; Moreira et al., 2020). Longitudinal yield behavior has been evaluated in cacao over longer time horizons, including multi-year studies of yield stability and temporal production patterns (Tahi et al., 2019; Dessauw et al., 2024). However, the genomic predictability of repeated-harvest disease trajectory targets, particularly for FPR and WBD, remains less well characterized.

A biological distinction between the two major *Moniliophthora* diseases may also influence predictability. WBD, caused by *M. perniciosa*, produces systemic symptoms in meristematic tissues including shoots and flower cushions, whereas FPR, caused by *M. roreri*, is largely pod-restricted and strongly shaped by local pod developmental stage and microclimatic conditions. These differences suggest that WBD-derived repeated-harvest summaries may retain a more stable accession-level component than FPR measurements.

Furthermore, practical breeding necessitates the simultaneous improvement of multiple, often negatively correlated traits, such as disease resistance and crop yield. Even when prediction is performed trait by trait, downstream breeding decisions still require integrative frameworks that balance competing agronomic objectives. Although multi-trait genomic models can improve prediction by leveraging covariance among traits (Garzón-Martínez et al., 2022; Semagn et al., 2022; Tang et al., 2024), practical candidate prioritization can also be approached using selection-index and Pareto-based frameworks. Such approaches allow breeders to rank accessions while balancing competing agronomic goals (Moeinizade et al., 2020).

In this study, we evaluated the genomic predictability of longitudinal disease and yield traits using repeated-harvest phenotypes from 102 cacao accessions genotyped across the Criollo and Matina reference genomes. We modeled temporal trajectories for healthy pod rate, FPR, and WBD to derive accession-specific baseline performance and temporal change. We subsequently evaluated prediction accuracy under random and relatedness-aware cross-validation scenarios. Finally, we tested the robustness of these predictions and applied a multi-trait selection index to identify candidate accessions that exhibit optimal combinations of temporal disease resilience and stable productivity.

## 2 Materials and methods

### 2.1 Plant materials and source data

This study used previously published cacao (*Theobroma cacao* L.) genotypic and phenotypic resources derived from the Agrosavia diversity panel, which included a larger genotyped collection and a phenotyped subset evaluated for productivity and disease-related traits across repeated harvest periods (Osorio-Guarín et al., 2020). The analytical workflow used accession metadata, repeated-harvest phenotype tables, sequencing summary tables, and two SNP datasets represented as VCF files corresponding to markers aligned to the Criollo and Matina cacao reference genomes (Osorio-Guarín et al., 2020).

Accession identifiers were standardized across metadata, phenotype, sequencing-summary, and VCF inputs before merging. Only accessions with both phenotype and genotype information were retained for genomic prediction analyses. The final prediction panel consisted of 102 phenotyped accessions, four ordered harvest periods, and 408 accession-by-harvest observations. The repeated-harvest panel included total pod number, healthy pod number, frosty pod rot severity, flower WBD severity, and branch WBD severity. A summary of the input datasets, trait definitions, marker sets, and derived prediction targets is provided in **Supplementary Data Sheet S1**.

### 2.2 Phenotype assembly and trait processing

Repeated-harvest phenotype records were reconstructed into a long-format panel with four ordered harvest periods. For each accession and harvest, the following traits were extracted: total pod number, healthy pod number, frosty pod rot severity, flower witches’ broom severity, and branch witches’ broom severity. Healthy pod rate was calculated as the proportion of healthy pods among total pods.

Because healthy pod rate is bounded between 0 and 1, it was transformed using an empirical logit with a pseudocount of 0.5 to stabilize variance. Frosty pod rot, flower witches’ broom, branch witches’ broom, and total pod number were transformed using $y^{\prime }=\log\left(1+x\right)$ to reduce right-skewness, stabilize variance, and accommodate zero values. Harvest time was encoded as an ordered variable and then standardized to mean 0 and variance 1 for longitudinal modeling.

In addition to the repeated-harvest panel, an accession-level “legacy” table was assembled from the accession totals for healthy pod rate, empirical-logit healthy pod rate, and the log-transformed disease and pod-count traits.

### 2.3 Longitudinal trajectory modeling

For each transformed trait, accession-specific temporal trajectories were modeled using linear mixed models for longitudinal data (Laird and Ware, 1982). The primary model included a fixed intercept and fixed time effect, with accession fitted as a random effect. The default specification attempted to estimate both accession-specific random intercepts and random slopes; when this was not estimable, a random-intercept model was used as a deterministic fallback.

The random-intercept/random-slope model was written as


$$y_{ij}={\text{β}}_{0}+{\text{β}}_{1}t_{j}+b_{0i}+b_{1i}t_{j}+\epsilon_{ij}$$

where *y_ij_* is the transformed phenotype of accession *i* at harvest *j*, *t_j_* is standardized harvest time, *β*_0_ and *β*_1_ are fixed intercept and time effects, b 0i ​ and b 1i ​ are accession-specific random intercept and slope deviations, and *ϵ_ij_* is the residual error. When the random-slope specification was not estimable, the fallback model was


$$y_{ij}={\text{β}}_{0}+{\text{β}}_{1}t_{j}+b_{0i}+\epsilon_{ij}$$

From each fitted model, accession-level trajectory features were extracted, including the accession-specific intercept, accession-specific slope, mean transformed phenotype across harvests, residual standard deviation, and the difference between the last and first harvest values. These accession-level summaries were used as downstream prediction targets.

Time-adjusted repeatability across the four harvests was estimated separately for each transformed trait. First, the fixed effect of time was removed using ordinary least squares to obtain time-adjusted trait values. Between-accession variance and mean within-accession residual variance were then estimated from these adjusted values to provide an operational summary of accession-level temporal consistency across four harvests. Repeatability was calculated as:


$$R=\frac{{\text{σ}}_{A}^{2}}{{\text{σ}}_{A}^{2}+{\text{σ}}_{e}^{2}/4}$$

where ${\text{σ}}_{A}^{2}$ represents the between-accession variance and ${\text{σ}}_{e}^{2}$ is the residual variance. This quantity was used as a measure of accession-level temporal consistency.

### 2.4 Genotype processing and reference-specific marker matrices

Genotypes were processed separately for the Criollo-based and Matina-based SNP datasets. VCF files were parsed into additive SNP dosages coded as 0, 1, or 2 copies of the alternative allele. Variants with multi-allelic genotype codes were excluded so that only biallelic dosage markers were retained.

For each reference framework, marker-level quality-control statistics were computed, including call rate, allele frequency, minor allele frequency (MAF), and heterozygosity. Variants were filtered using MAF ≥ 0.05 and call rate ≥ 0.95. Missing dosages among retained markers were imputed using the marker mean. Marker dosages were then standardized to mean 0 and unit variance across accessions.

Sample-level quality-control summaries were also computed, including sample call rate and heterozygosity. Prediction analyses were restricted to the phenotyped subset after alignment of accession order between phenotype and genotype tables.

After quality filtering, the Criollo marker matrix contained 9,003 SNPs spanning approximately 320.9 Mb with a mean inter-marker spacing of 35.7 kb, whereas the Matina marker matrix contained 8,131 SNPs spanning approximately 329.6 Mb with a mean inter-marker spacing of 40.8 kb. Marker-distribution summaries, including median spacing, 90th-percentile spacing, and maximum gap size, are provided in **Supplementary Data Sheet S1**. Missingness among retained markers in the phenotyped subset was zero after filtering; therefore, marker-mean imputation did not alter retained genotype dosages. As a sensitivity check, KNN imputation with *k* = 5 was also evaluated and yielded identical prediction results.

### 2.5 Principal components and relatedness-aware blocking

Population structure summaries were obtained by principal component analysis (PCA) on the standardized marker matrix (Price et al., 2006). The first 10 principal components were retained for downstream baseline prediction.

To reduce inflation of prediction accuracy caused by relatedness leakage between training and test sets, relatedness-aware cross-validation blocks were constructed from the standardized marker matrix by hierarchical clustering with Ward’s minimum-variance criterion using Euclidean distances (Ward, 1963). In the primary analysis, accessions were partitioned into approximately five genotype-derived relatedness blocks. In robustness analyses, the number of blocks was varied from 4 to 7. In addition, alternative genotype-derived block definitions were evaluated using *k*-means clustering on the first 10 marker principal components and *k*-means clustering on eigenvectors of the marker-derived relationship matrix. These alternative blocking analyses were used to assess whether the WBD prediction hierarchy depended on the original Ward-clustering definition.

To contextualize the small-*n*/high-*p* prediction setting, we summarized the eigenvalue spectrum of the marker-derived relationship matrix. Because pedigree records and recombination-map information were not available, formal estimates of effective population size or independent chromosome segments were not attempted; instead, an eigenvalue-based effective marker dimensionality was calculated as ${\left(\sum_{l}{\text{λ}}_{l}\right)}^{2}/\sum_{l}{\text{λ}}_{l}^{2}$ where *λ_l_* are the eigenvalues of the relationship matrix.

### 2.6 Genomic prediction

Prediction targets included both accession-level legacy phenotypes and trajectory-derived longitudinal summaries. The main prediction set comprised healthy pod rate, frosty pod rot, flower witches’ broom, and branch witches’ broom legacy traits, together with accession-specific intercept and slope estimates for the transformed longitudinal traits. All targets were standardized before model fitting.

Three primary prediction models were evaluated. The first was a null mean predictor. The second was a ridge-regression baseline fitted on the genotype principal components, which served as a low-dimensional population-structure baseline (Hoerl and Kennard, 1970; Price et al., 2006). The third was a genomic best linear unbiased prediction (GBLUP)-like kernel model implemented as kernel ridge regression using the marker-derived relationship kernel:


$$K=\frac{XX^{T}}{m}$$

where *X* is the standardized marker matrix and *m* is the number of markers (Meuwissen et al., 2001; VanRaden, 2008; Endelman, 2011).

In the primary workflow, random cross-validation used 20 repeats of 5-fold cross-validation. Relatedness-aware validation used group-based cross-validation in which the genotype-derived relatedness blocks were held out one block at a time. For ridge-based models, shrinkage parameters were selected from a fixed grid by internal cross-validation. For the kernel model, the penalty parameter was selected by inner cross-validation on the training set.

Prediction accuracy was summarized using Pearson correlation, Spearman rank correlation, root mean squared error (RMSE), mean absolute error (MAE), and coefficient of determination (*R*^2^). For repeated random cross-validation, mean performance, standard deviation, and percentile-based intervals were calculated across repeats.

### 2.7 Empirical permutation testing

To assess whether relatedness-blocked prediction performance exceeded chance expectation, empirical permutation tests were performed for the GBLUP model under the blocked-validation setting. Trait labels were permuted 10,000 times for each reference-by-target test. Empirical *p*-values were computed as


$$p_{\text{emp}}=\frac{1+b}{1+m}$$

where *b* is the number of permuted statistics at least as large as the observed statistic and *m* = 10,000 is the number of permutations (Phipson and Smyth, 2010). To account for the 24 reference-by-target tests, empirical *p*-values were adjusted using the Benjamini–Hochberg false-discovery-rate procedure. For computational consistency, permutation testing used the penalty parameter selected from the corresponding blocked-validation workflow rather than independently re-tuning the penalty within each permuted dataset.

### 2.8 Trajectory robustness analysis

A dedicated robustness layer was used to test whether accession-level temporal summaries depended strongly on the choice of trajectory model. For each transformed trait, three trajectory estimators were compared: (i) a random-intercept mixed model; (ii) a random-intercept/random-slope mixed model; and (iii) a per-accession ordinary least squares model fit separately within each accession.

For the mixed models, restricted maximum likelihood estimation was attempted using multiple optimizers. For each model specification, accession-level intercepts, slopes, and residual standard deviations were extracted. Robustness was then summarized in two ways: first, by pairwise Pearson and Spearman correlations of accession estimates across model specifications; and second, by overlap among the top 10 favorable accessions identified by each specification. Trait-specific consensus trajectory summaries were calculated as medians across model specifications for descriptive robustness assessment.

### 2.9 Selection index and breeding prioritization

A multi-trait selection index was calculated from standardized accession-level trajectory summaries.

The selection index for accession *i* was defined as


$$I_{i}=\sum_{k}w_{k}z_{ik}$$

where *z_ik_* ​ is the standardized value of trajectory component k for accession *i*, and w *k* ​ is the corresponding component weight. Positive weights favored higher healthy-pod performance, whereas negative weights penalized higher disease burden, increasing disease trajectories, and residual temporal variability.

Healthy pod intercept and slope were given positive weights, whereas disease intercepts, disease slopes, and within-accession residual variability were penalized. In the default index, healthy pod intercept, slope, and residual standard deviation received weights of +1.0, +0.5, and -0.25, respectively. For frosty pod rot, flower witches’ broom, and branch witches’ broom, intercept, slope, and residual standard deviation received weights of -1.0, -0.5, and -0.25, respectively. The weighted standardized components were summed to obtain an accession-level selection index, and accessions were ranked from highest to lowest.

In parallel, a Pareto-based multiobjective screen was applied using healthy pod performance as a favorable objective and disease intercepts as unfavorable objectives. Accessions were classified as Pareto-optimal when no other accession was equal or better for all objectives and strictly better for at least one objective.

### 2.10 Selection-index sensitivity analysis

To test whether accession rankings were robust to the exact weighting scheme, the selection index was recalculated under both deterministic and random perturbations of the weights. Deterministic alternatives emphasized, in turn, intercept-only effects, equal weighting of intercept and slope, stronger weighting of temporal stability, stronger weighting of slope, witches’ broom priority, productivity priority, and balanced disease weighting. In addition, 250 random schemes were generated by multiplying each non-zero weight by a random factor sampled from a uniform distribution between 0.8 and 1.2.

For each scheme, accessions were re-ranked and summarized by mean rank, median rank, rank standard deviation, frequency of appearing in the top 1, top 5, and top 10, and frequency of being Pareto-optimal. Pairwise rank concordance among deterministic schemes was quantified using Pearson and Spearman correlations.

### 2.11 Prediction stress testing

A second robustness analysis evaluated the sensitivity of genomic prediction to the definition of relatedness blocks and to model class. Under block settings of 4, 5, 6, and 7 genotype-derived groups, blocked cross-validation was repeated for the null mean predictor, the principal-component ridge model, an rrBLUP-like ridge regression fitted directly on the marker matrix, and the GBLUP-like kernel model (Hoerl and Kennard, 1970; Meuwissen et al., 2001; Endelman, 2011). In this stress-test analysis, the ridge-penalty grid was expanded to cover a wider range of values.

For each target and model, overall accuracy, fold-wise accuracy, and observed-versus-predicted pairs were stored. Model-rank stability across block definitions was summarized by mean model rank, rank standard deviation, frequency of being the best-performing model, and the range of observed Pearson correlations across block settings. Target-level robustness was summarized by the mean, standard deviation, minimum, and maximum Pearson correlations across block settings. Randomized procedures used throughout the prediction and robustness analyses were controlled by a fixed seed to ensure reproducibility. Expanded derived analytical outputs supporting the main prediction and robustness results are provided in **Supplementary Data Sheet S1**.

## 3 Results

### 3.1 Repeated-harvest phenotypes revealed stable accession-level temporal structure

**Figure 1:**
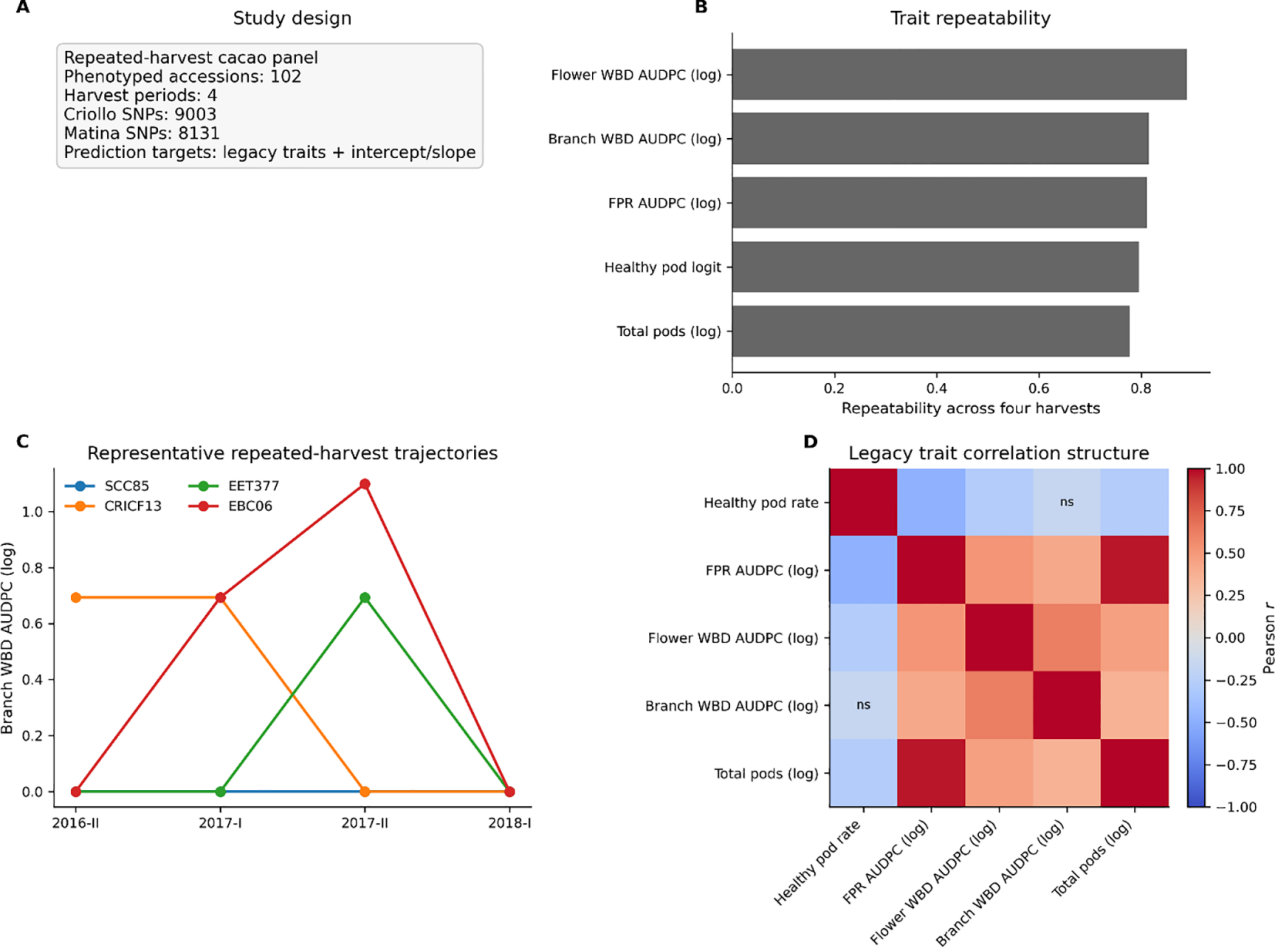
Study overview and repeated-harvest phenotype structure. **(A)** Summary of the repeated-harvest cacao panel, including the number of phenotyped accessions, harvest periods, and Criollo- and Matina-based SNP sets. **(B)** Repeatability estimates across four harvests for the transformed traits. **(C)** Representative repeated-harvest trajectories illustrating accession-specific temporal patterns. **(D)** Correlation structure among legacy traits, with non-significant pairwise relationships labeled as ns.

The repeated-harvest cacao panel comprised 102 phenotyped accessions evaluated across four harvest periods, together with two marker frameworks consisting of 9,003 Criollo-based SNPs and 8,131 Matina-based SNPs after quality filtering (**Figure 1A**). These marker sets covered approximately 320.9 Mb in the Criollo framework and 329.6 Mb in the Matina framework, with mean inter-marker spacing of 35.7 kb and 40.8 kb, respectively. Prediction targets included both legacy trait summaries and trajectory-derived intercept and slope estimates. Across the four harvest periods, accession-level temporal repeatability was consistently high, indicating that the observed trait variation retained a substantial accession-level component rather than being driven primarily by transient within-panel fluctuation (**Table 1**). Repeatability was highest for flower WBD AUDPC (log), followed by branch WBD AUDPC (log), FPR AUDPC (log), healthy pod logit, and total pods (log) (**Figure 1B**; **Table 1**). Although the more complex random-intercept/random-slope formulation did not fully converge for flower WBD AUDPC (log), the trait nevertheless showed the strongest accession-level temporal repeatability in the simpler repeatability analysis. Thus, WBD-related traits showed the strongest accession-level consistency over time, supporting their use as longitudinal prediction targets while motivating a later robustness check across trajectory estimators.

**Table 1:** Repeatability of repeated-harvest cacao traits.

Trait	Repeatability across 4 harvests	Slope *p*-value	Random-slope model converged
Flower WBD AUDPC (log)	0.889	4.27E-01	No
Branch WBD AUDPC (log)	0.816	3.61E-01	Yes
FPR AUDPC (log)	0.812	4.72E-46	Yes
Healthy pod logit	0.796	6.18E-05	Yes
Total pods (log)	0.777	2.06E-36	Yes

Time-adjusted repeatability estimates for transformed repeated-harvest traits across four harvest periods. Higher values indicate stronger accession-level temporal consistency after accounting for harvest effects. Traits are shown in the same transformed scale used for downstream trajectory modeling and genomic prediction.

Representative repeated-harvest trajectories further showed that accessions differed not only in average branch WBD burden but also in the timing and magnitude of temporal fluctuation across harvests (**Figure 1C**). Some accessions remained nearly flat across all four harvest periods, whereas others displayed more pronounced transient peaks, particularly around 2017-II, before returning to lower values by 2018-I. These contrasting patterns indicate that repeated-harvest phenotypes capture both baseline disease status and accession-specific temporal instability, supporting the use of trajectory-derived summaries rather than simple across-harvest totals alone.

Legacy trait relationships were also biologically coherent (**Figure 1D**). The two WBD-related variables were positively correlated with one another, whereas healthy pod rate showed weak or non-significant relationships with branch WBD. This overall structure suggests that WBD-related traits capture a partially shared component of disease response that is distinct from simple pod productivity, while also motivating the separation of baseline and temporal components in downstream genomic prediction analyses.

### 3.2 WBD-related longitudinal targets were the most genomically predictable

**Figure 2:**
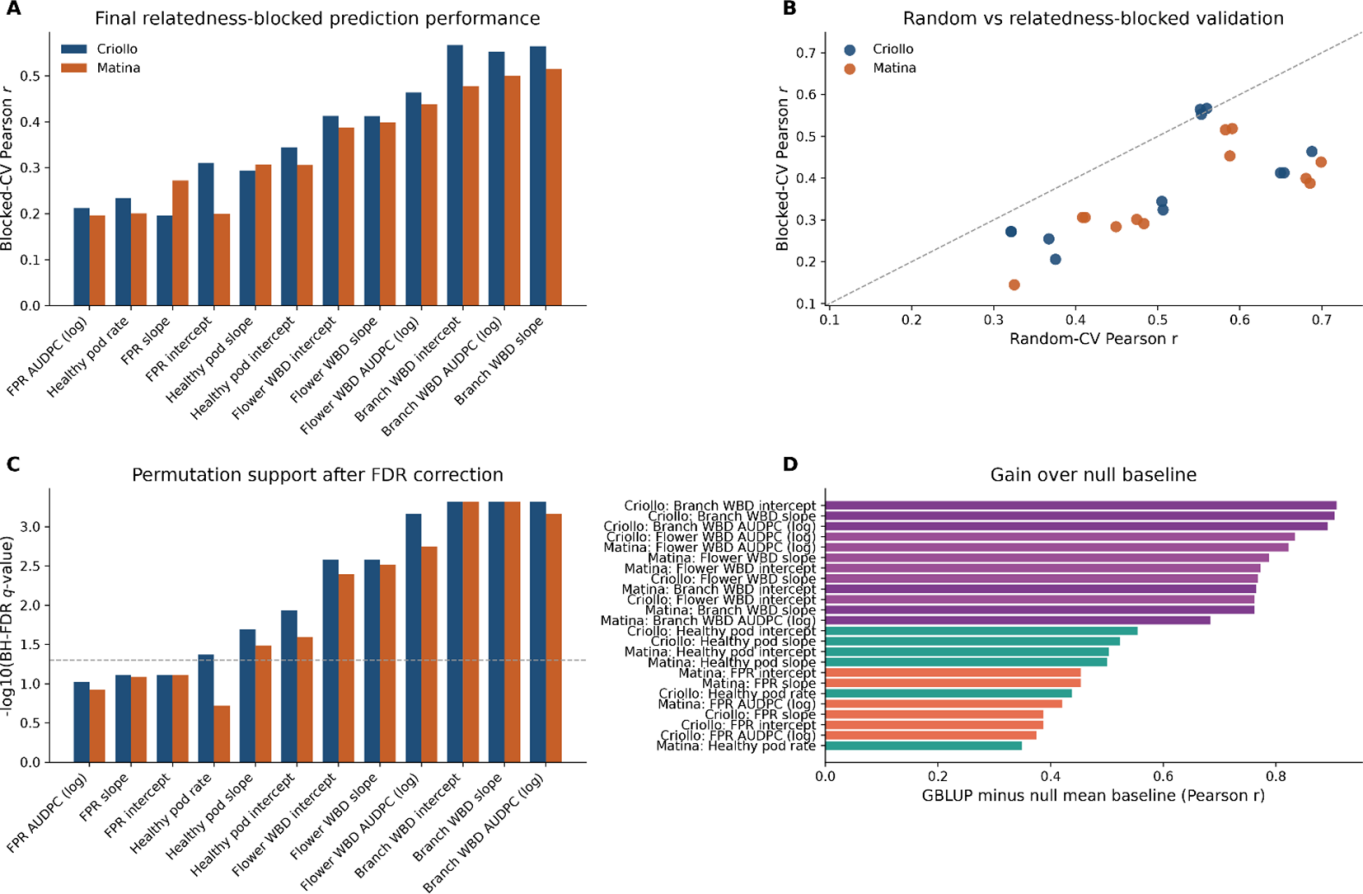
Final prediction performance and support for WBD-related targets. **(A)** Final blocked-cross-validation prediction performance for each legacy and trajectory-derived target under Criollo and Matina marker frameworks. **(B)** Comparison of random cross-validation and relatedness-blocked cross-validation, showing the expected reduction in performance under stricter validation. **(C)** Empirical permutation support summarized after 10,000 label permutations and Benjamini–Hochberg false-discovery-rate correction across 24 reference-by-target tests. **(D)** Improvement of the GBLUP model over the null mean baseline, expressed as the increase in Pearson correlation. WBD-related targets, especially branch and flower WBD summaries, consistently showed the strongest predictive signal.

Genomic prediction analyses revealed a clear hierarchy among targets under the stricter relatedness-blocked validation scheme (**Table 2**). Under this setting, WBD-related traits consistently occupied the highest-performing tier under relatedness-blocked cross-validation (**Figure 2A**; **Table 2**). In both reference frameworks, branch WBD intercept, branch WBD slope, branch WBD AUDPC (log), flower WBD AUDPC (log), flower WBD slope, and flower WBD intercept outperformed healthy pod rate and most FPR-derived targets. Although Criollo generally produced slightly higher peak performance than Matina for the strongest WBD targets, the overall ranking of targets was similar across the two marker frameworks. In contrast, healthy pod rate and FPR AUDPC (log) remained in the lower-performing range, with blocked-cross-validation Pearson correlations clustered near the bottom of the target set.

**Table 2:** Final genomic prediction metrics for legacy and trajectory-derived targets.

Reference	Trait class	Target	Blocked-CV Pearson *r*	Permutation observed Pearson *r*	Empirical permutation *p*	BH-FDR *q*-value	FDR-supported (*q* < 0.05)
Criollo	Branch WBD	Branch WBD intercept	0.567	0.567	0.0001	0.0005	Yes
Criollo	Branch WBD	Branch WBD slope	0.564	0.564	0.0001	0.0005	Yes
Criollo	Branch WBD	Branch WBD AUDPC (log)	0.553	0.538	0.0001	0.0005	Yes
Criollo	Flower WBD	Flower WBD AUDPC (log)	0.464	0.464	0.0002	0.0007	Yes
Criollo	Flower WBD	Flower WBD intercept	0.413	0.413	0.0010	0.0026	Yes
Criollo	Flower WBD	Flower WBD slope	0.412	0.412	0.0011	0.0026	Yes
Criollo	Healthy pod	Healthy pod intercept	0.344	0.344	0.0063	0.0116	Yes
Criollo	FPR	FPR intercept	0.310	0.236	0.0600	0.0775	No
Criollo	Healthy pod	Healthy pod slope	0.294	0.324	0.0119	0.0204	Yes
Criollo	Healthy pod	Healthy pod rate	0.234	0.271	0.0302	0.0426	Yes
Criollo	FPR	FPR AUDPC (log)	0.212	0.214	0.0873	0.0952	No
Criollo	FPR	FPR slope	0.196	0.236	0.0638	0.0775	No
Matina	Branch WBD	Branch WBD slope	0.516	0.516	0.0001	0.0005	Yes
Matina	Branch WBD	Branch WBD AUDPC (log)	0.500	0.500	0.0002	0.0007	Yes
Matina	Branch WBD	Branch WBD intercept	0.478	0.519	0.0001	0.0005	Yes
Matina	Flower WBD	Flower WBD AUDPC (log)	0.438	0.438	0.0006	0.0018	Yes
Matina	Flower WBD	Flower WBD slope	0.399	0.399	0.0014	0.0031	Yes
Matina	Flower WBD	Flower WBD intercept	0.388	0.388	0.0020	0.0040	Yes
Matina	Healthy pod	Healthy pod slope	0.307	0.296	0.0217	0.0325	Yes
Matina	Healthy pod	Healthy pod intercept	0.306	0.306	0.0159	0.0254	Yes
Matina	FPR	FPR slope	0.272	0.231	0.0718	0.0820	No
Matina	Healthy pod	Healthy pod rate	0.201	0.137	0.1901	0.1901	No
Matina	FPR	FPR intercept	0.200	0.231	0.0646	0.0775	No
Matina	FPR	FPR AUDPC (log)	0.196	0.196	0.1145	0.1195	No

Summary of relatedness-blocked GBLUP prediction performance across legacy and trajectory-derived targets under Criollo and Matina marker frameworks. Reported metrics include the final blocked-cross-validation Pearson correlation, the observed statistic used in the high-resolution permutation test, the empirical *p*-value based on 10,000 label permutations, and the Benjamini–Hochberg false-discovery-rate *q*-value across the 24 reference-by-target tests. Because the permutation workflow used a fixed penalty parameter selected from the corresponding blocked-validation workflow, the permutation-test observed statistic may differ slightly from the final reported blocked-cross-validation correlation.

As expected, performance under random cross-validation was generally higher than performance under relatedness-blocked cross-validation, indicating that conventional random partitioning captures some signal attributable to relatedness between training and test samples (**Figure 2B**). However, the strongest WBD-related targets remained clearly predictive even under blocked validation. High-resolution permutation testing reinforced this conclusion. After 10,000 label permutations and Benjamini–Hochberg correction across 24 reference-by-target tests, branch and flower WBD-derived targets retained the strongest empirical support, whereas FPR-derived targets did not remain significant at FDR< 0.05 in either reference framework (**Figure 2C**; **Table 2**). Comparison against the null mean baseline led to the same conclusion, because the largest gains over baseline were concentrated among branch and flower WBD targets (**Figure 2D**). Notably, this hierarchy was broadly preserved across both reference frameworks, although some targets, particularly healthy pod rate and selected non-WBD components, showed greater reference sensitivity than the strongest WBD-derived targets. These results identify WBD-related longitudinal traits as the most robustly predictable component of the phenotype set.

### 3.3 Trajectory summaries and prediction results were robust to modeling choices

**Figure 3:**
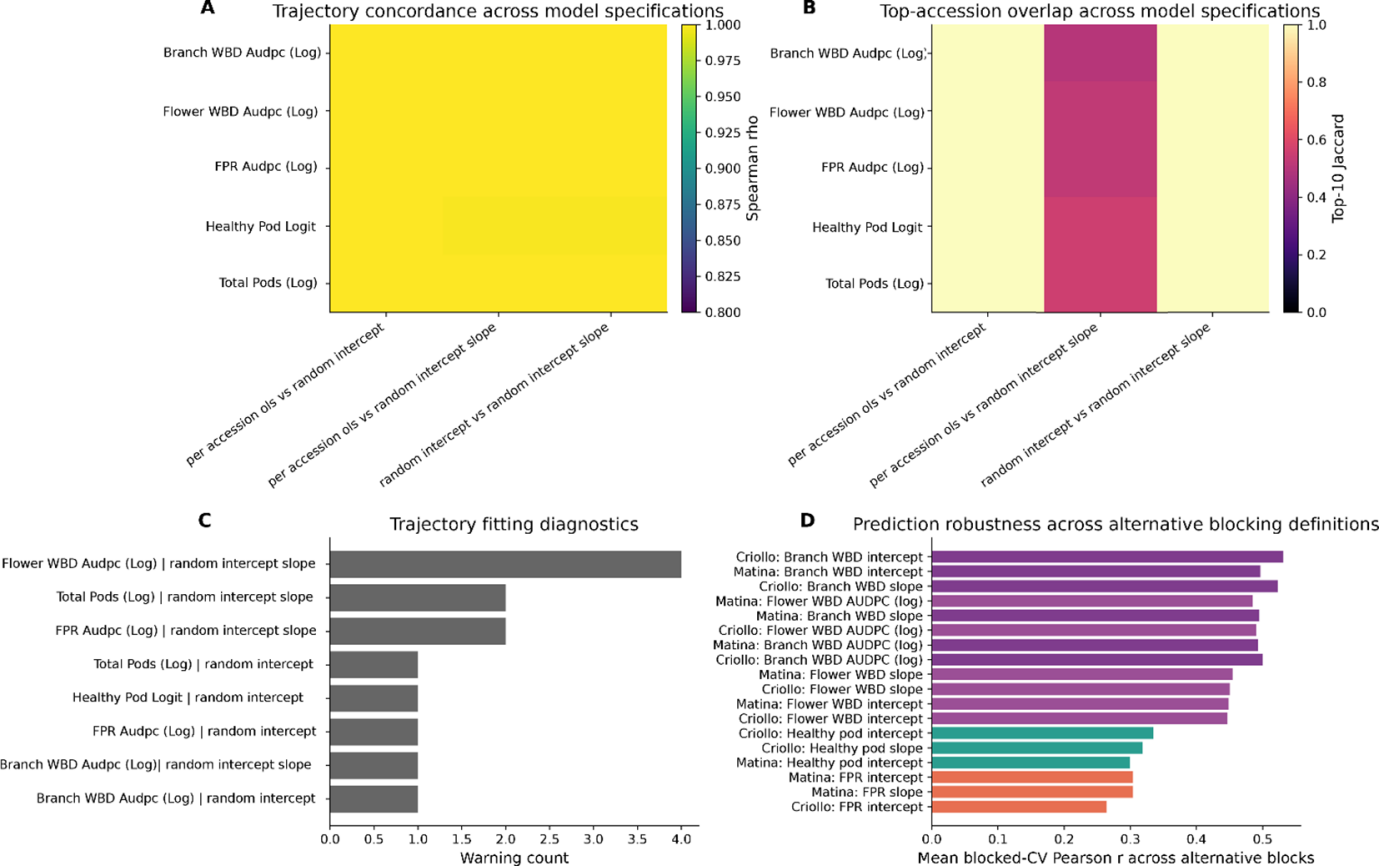
Robustness of trajectory estimation and prediction under stress testing. **(A)** Concordance of accession-level trajectory summaries across model specifications. **(B)** Overlap among top-ranked accessions across model specifications. **(C)** Trait-specific trajectory fitting diagnostics, summarized as warning counts. **(D)** Mean prediction performance across alternative relatedness-blocking definitions, including Ward marker-distance clustering, *k*-means clustering on marker principal components, and *k*-means clustering on relationship-matrix eigenvectors. Branch and flower WBD targets remained concentrated among the strongest-performing outcomes across blocking definitions.

Because longitudinal inference can depend on how accession-specific trajectories are estimated, we next evaluated robustness across model specifications. Trajectory summaries derived from per-accession ordinary least squares, random-intercept mixed models, and random-intercept/random-slope mixed models were highly concordant across all traits (**Figure 3A**). Likewise, overlap among top-ranked accessions was high across specifications (**Figure 3B**), indicating that accession prioritization was not strongly dependent on one specific trajectory estimator. These results suggest that the accession-level longitudinal targets used for genomic prediction were stable summaries of repeated-harvest behavior rather than artifacts of one modeling choice.

Some fitting warnings were observed, especially for the more complex random-intercept/random-slope models, with the largest warning count occurring for flower WBD AUDPC (log) (**Figure 3C**). However, these warnings were not accompanied by major changes in cross-model accession concordance, top-accession overlap, or the downstream prediction hierarchy. This pattern supports the view that the main prediction results were not driven by one unstable trajectory specification. Stress testing across different relatedness-block definitions also yielded a consistent pattern: the highest mean Pearson correlations across block settings remained concentrated in branch WBD and, secondarily, flower WBD targets, particularly under GBLUP and related ridge-based models (**Figure 3D**). Thus, the central conclusion that WBD is the most genomically predictable phenotype class was stable across both trajectory-model choice and block-setting variation. This pattern also persisted when relatedness blocks were defined using alternative genotype-derived strategies, including *k*-means clustering on marker principal components and *k*-means clustering on relationship-matrix eigenvectors. Across 12 blocking tests per reference, branch and flower WBD targets remained concentrated among the highest-ranked prediction outcomes, whereas FPR and healthy-pod targets were generally lower-ranked (**Supplementary Figure 1**; **Supplementary Data Sheet S1**). Because no missing genotypes remained among retained markers in the phenotyped subset after QC, mean imputation and KNN imputation produced identical prediction results, indicating that the reported prediction hierarchy was not driven by the imputation procedure (**Supplementary Figure 2**; **Supplementary Data Sheet S1**). Expanded robustness summaries, including model concordance, top-accession overlap, warning diagnostics, and stress-test outputs, are provided in **Supplementary Data Sheet S1**.

### 3.4 Multi-trait ranking identified a stable breeding shortlist

**Figure 4:**
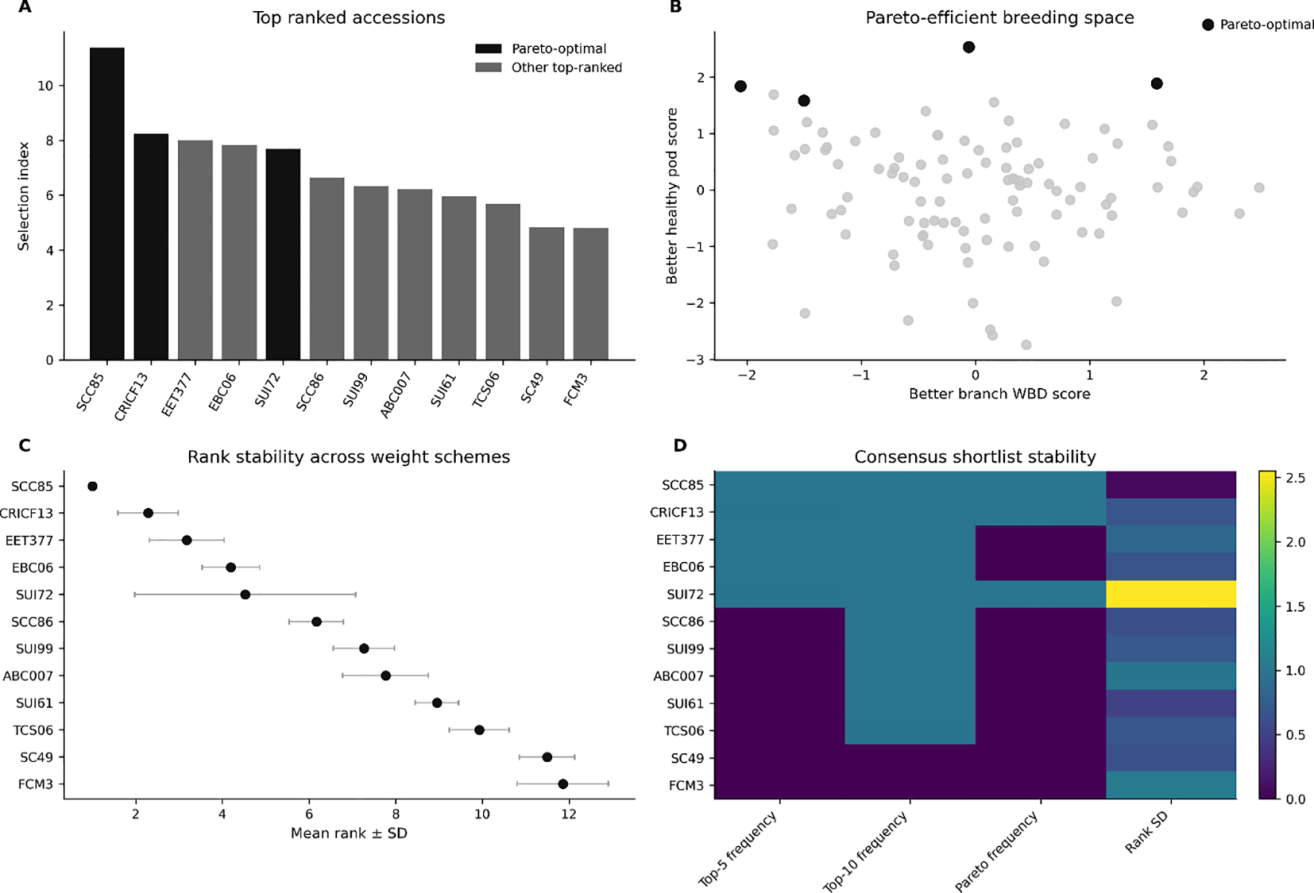
Multi-trait ranking and stability of the breeding shortlist. **(A)** Top-ranked accessions based on the multi-trait selection index, with Pareto-optimal accessions highlighted. **(B)** Pareto-efficient breeding space defined by healthy pod and branch WBD components. **(C)** Mean rank and standard deviation across alternative weighting schemes, illustrating shortlist stability. **(D)** Heatmap summarizing top-5 frequency, top-10 frequency, Pareto frequency, and rank standard deviation for the leading candidates.

To translate prediction-oriented results into breeding-oriented prioritization, we constructed a multi-trait selection index and complemented it with Pareto-based screening (**Figure 4A**). The highest-ranked accessions were SCC85, CRICF13, EET377, EBC06, and SUI72, followed by SCC86, SUI99, ABC007, SUI61, TCS06, SC49, and FCM3 (**Table 3**). Across the full accession set, four accessions were identified as Pareto-optimal in the two-dimensional space defined by better healthy pod score and better branch WBD score (**Figure 4B**). Of these, three also appeared in the rank-stable top shortlist reported in **Figure 4A** and **Table 3**, whereas the remaining Pareto-optimal accession fell outside the final consensus shortlist under the broader multi-trait ranking criteria.

**Table 3:** Consensus breeding shortlist from multi-trait stability analyses.

Accession	Selection index	Rank	Pareto-optimal	Mean rank across weight schemes	Rank SD	Top-5 frequency	Top-10 frequency	Pareto frequency
**SCC85**	**11.390**	**1**	**Yes**	**1.004**	**0.062**	**100.0%**	**100.0%**	**100.0%**
**CRICF13**	**8.238**	**2**	**Yes**	**2.287**	**0.697**	**99.2%**	**100.0%**	**100.0%**
**EET377**	**7.998**	**3**	**No**	**3.178**	**0.864**	**99.6%**	**100.0%**	**0.0%**
**EBC06**	**7.822**	**4**	**No**	**4.198**	**0.663**	**99.6%**	**100.0%**	**0.0%**
**SUI72**	**7.687**	**5**	**Yes**	**4.527**	**2.549**	**99.2%**	**99.6%**	**100.0%**
SCC86	6.638	6	No	6.171	0.625	0.8%	99.6%	0.0%
SUI99	6.332	7	No	7.267	0.707	0.4%	99.6%	0.0%
ABC007	6.213	8	No	7.767	0.990	0.8%	99.6%	0.0%
SUI61	5.950	9	No	8.950	0.500	0.0%	100.0%	0.0%
TCS06	5.684	10	No	9.930	0.691	0.0%	98.8%	0.0%
SC49	4.842	11	No	11.492	0.638	0.0%	0.4%	0.0%
FCM3	4.806	12	No	11.857	1.054	0.0%	0.4%	0.0%
PA46	4.699	13	No	12.783	0.648	0.0%	0.0%	0.0%
UF273	4.253	14	No	14.636	1.713	0.4%	0.4%	0.0%
TCS19	4.177	15	No	14.950	1.088	0.0%	0.4%	0.0%
EBC09	4.058	16	No	15.519	1.026	0.0%	0.0%	0.0%
DONDIEGO	3.584	17	No	17.190	0.817	0.0%	0.0%	0.0%
TCS36	3.224	18	No	18.027	0.820	0.0%	0.0%	0.0%
PA121	2.931	19	No	19.829	1.333	0.0%	0.0%	0.0%
TCS39	2.859	20	No	20.132	2.007	0.0%	0.0%	0.0%

Rank-stable accession shortlist based on multi-trait selection-index analysis and Pareto-based prioritization. The table summarizes the accessions that remained most consistently favorable across alternative weighting schemes and complementary prioritization criteria. Bold values indicate the top five accessions according to the default multi-trait selection-index ranking.

Notably, this shortlist was not an artifact of a single hand-tuned weighting scheme. Rank stability analyses showed that the leading accessions remained near the top across multiple weighting schemes (**Figure 4C**), and the consensus-stability heatmap indicated repeated retention of the top candidates in the top-5, top-10, and Pareto-prioritized sets (**Figure 4D**). SCC85 was especially stable, with almost no movement in rank across weighting schemes, whereas CRICF13, EET377, EBC06, and SUI72 also remained consistently competitive (**Table 3**). These results support the interpretation that the breeding shortlist reflects a stable multi-trait signal rather than a fragile ranking driven by one arbitrary index definition.

### 3.5 WBD-related prediction signals were reproducible across reference-genome frameworks

**Figure 5:**
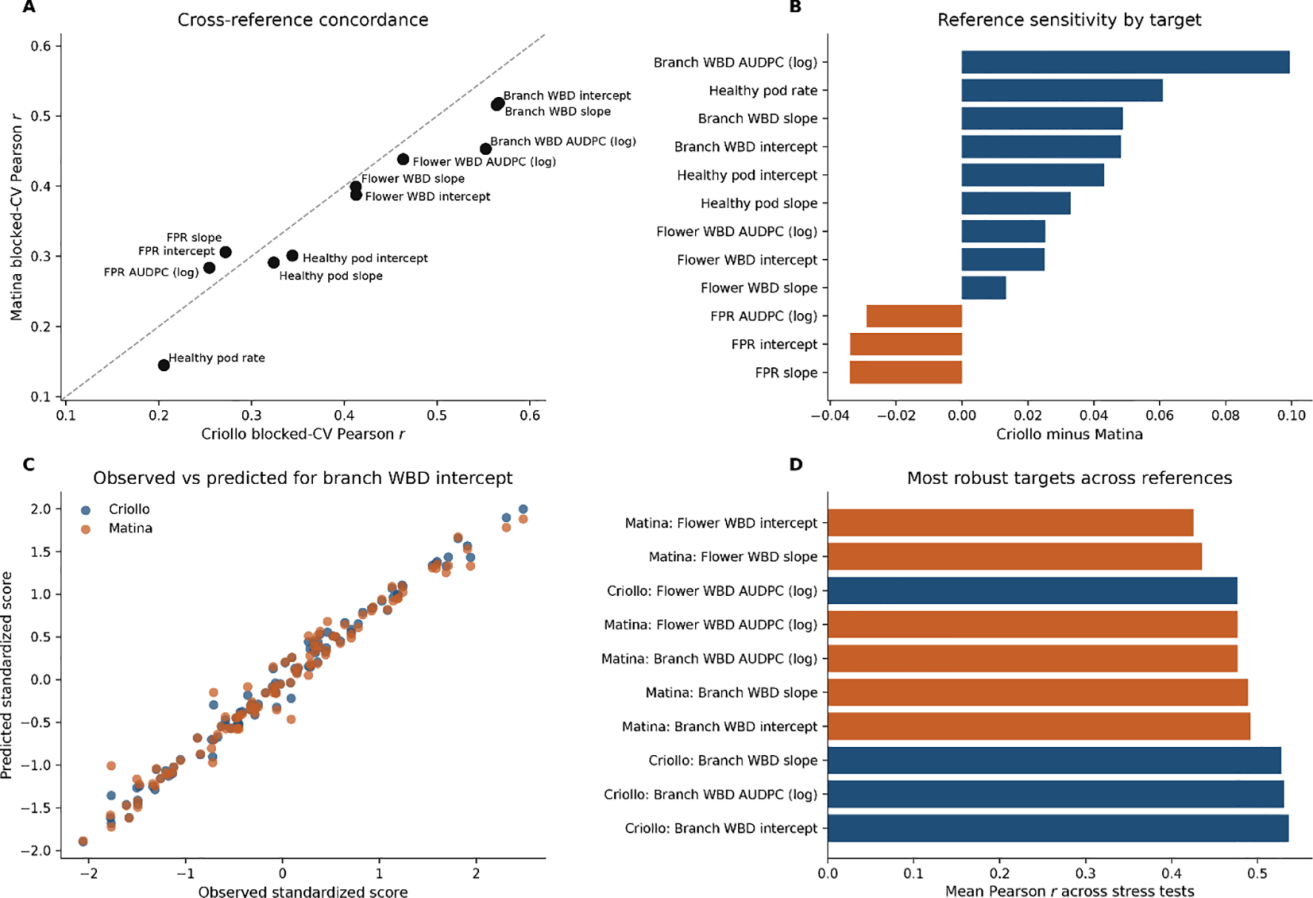
Cross-reference reproducibility of genomic prediction targets. **(A)** Concordance of blocked-cross-validation performance between Criollo and Matina marker frameworks. **(B)** Reference sensitivity of each target, expressed as the difference in prediction performance between the two references. **(C)** Observed versus predicted standardized scores for branch WBD intercept under both references. **(D)** Most robust targets across references based on stress-test summaries. WBD-derived targets, especially branch WBD components, were the most reproducible across marker frameworks.

Cross-reference comparisons showed that the strongest genomic prediction signals were reproducible between the Criollo and Matina marker frameworks. In the concordance plot, branch WBD intercept, branch WBD AUDPC (log), and flower WBD AUDPC (log) clustered among the strongest-performing targets in both references (**Figure 5A**). Reference-sensitivity analysis likewise showed that the largest positive Criollo-minus-Matina differences were concentrated in branch WBD and healthy pod components, whereas FPR intercept and FPR slope tended to show small negative differences, indicating slightly better relative performance under Matina for those specific targets (**Figure 5B**). Overall, however, the most robust targets across the two references were dominated by branch WBD and flower WBD summaries rather than by healthy pod rate or FPR.

The observed-versus-predicted comparison for branch WBD intercept further illustrated this reproducibility, because predictions from both reference frameworks broadly tracked the standardized observed values across accessions (**Figure 5C**). When robustness across stress tests was summarized directly, branch WBD intercept, branch WBD AUDPC (log), and branch WBD slope occupied the top positions, followed by flower WBD-derived targets (**Figure 5D**). These cross-reference results strengthen the main conclusion of the study: repeated-harvest WBD-related temporal targets were the most predictable within each marker framework and the most concordant across the two reference-genome marker systems evaluated here. These cross-reference comparisons were used to evaluate analytical concordance across marker frameworks rather than to infer biological differences between reference genomes.

## 4 Discussion

A central contribution of this study is the application of a longitudinal genomic-prediction framework to repeated-harvest cacao disease traits. In perennial crops, trait expression is a continuous process shaped by accumulated environmental exposures and growth cycles (Moreira et al., 2020). Single-time-point measurements often confound true genetic merit with transient environmental noise. Utilizing repeated-harvest data allows for the separation of baseline performance from temporal progression, an approach shown to improve the estimation of genotypic values and stability in other perennial species (Seyum et al., 2022; Fernandes Filho et al., 2025). The decomposition of longitudinal phenotypes into intercepts and slopes offers a biological interpretation alongside statistical utility. In this framework, the intercept can be interpreted as a proxy for baseline susceptibility or constitutive defense status, whereas the slope reflects the direction and relative rate of temporal disease change across harvests. The high repeatability observed for disease and yield traits across four harvests in this study supports the utility of longitudinal descriptors over simple aggregate means for cacao breeding.

Prediction accuracies varied significantly among traits, with WBD-related longitudinal targets consistently outperforming those for FPR and healthy pod rate. This disparity likely reflects fundamental differences in the epidemiology and infection biology of the two *Moniliophthora* species. *M. perniciosa* is a systemic pathogen that infects meristematic tissues, inducing hypertrophic growth in shoots, flower cushions, and pods (Griffith et al., 2003; Marelli et al., 2009). Consequently, WBD burden can accumulate within the host architecture over time, which may help generate a more stable accession-specific phenotypic signature than that observed for pod-restricted disease outcomes. In contrast, *M. roreri* exclusively infects actively growing pods (Phillips-Mora et al., 2007; Evans, 2016). Infection success and subsequent disease severity for FPR are highly contingent on the coincidence of susceptible pod developmental stages with specific microclimatic variables, such as ambient humidity and rainfall (Ploetz, 2016). This pronounced environmental dependency likely introduces substantial non-genetic variance into FPR measurements, thereby limiting genomic predictability relative to WBD-derived targets, even though FPR itself remained reasonably repeatable across harvests in this panel.

The present results also clarify the functional distinction between locus discovery and genomic prediction in cacao. Previous genome-wide association studies (GWAS) on this population identified candidate regions associated with disease resistance (Osorio-Guarín et al., 2020; Ahn et al., 2025). While GWAS is effective for identifying specific genomic regions of large effect, it often fails to capture the full extent of heritable variation for complex traits due to the highly polygenic nature of resistance and the small effect sizes of individual loci, a phenomenon well-documented across forest and crop species (Kainer et al., 2015; Bian and Holland, 2017; Müller et al., 2017). Genomic prediction models, by using genome-wide marker information distributed across many loci, account for this polygenic background (Wolc and Dekkers, 2022). The robust prediction accuracies observed here, particularly for WBD under relatedness-blocked cross-validation, are consistent with a distributed genetic architecture rather than one dominated by only a few major loci. Direct locus-by-locus comparison with previous GWAS signals was not the primary objective of the present analysis because the main prediction model used genome-wide kernel relationships rather than marker-level association testing. Therefore, the present results should be interpreted as evidence for genome-wide predictive signal rather than as fine mapping of causal loci. Future work combining marker-effect decomposition, local genomic relationship mapping, and independent validation panels will be needed to evaluate overlap between predictive genomic regions and previously reported GWAS candidates.

Evaluating model performance through relatedness-blocked cross-validation provided a rigorous assessment of predictive utility. Accuracy generally declines when relatedness between training and testing sets is minimized, as the model cannot rely on extensive shared haplotypes (Müller et al., 2017). The persistence of the predictive signal for branch and flower WBD targets under blocked validation demonstrates that the genomic models capture broad additive genetic effects applicable under stricter relatedness-aware validation settings. Furthermore, these targets exhibited high stability across alternative modeling algorithms, mixed-model specifications, and reference genome frameworks (Criollo and Matina). The stability of WBD predictive signals across both reference frameworks suggests that the underlying genomic signal is comparatively robust to marker-framework choice and is less reference-sensitive than the corresponding signals for healthy pod rate or some FPR-derived targets. Accordingly, the WBD predictive signal is unlikely to be an artifact of a specific analytical choice and instead appears to reflect a reproducible accession-level genomic component.

The multi-trait selection index provides a practical way to apply these predictive models to breeding decisions. Disease resistance must be balanced with productivity to ensure commercial viability. Such index-based prioritization allows breeders to simultaneously optimize these competing objectives and manage negatively correlated traits efficiently (Moeinizade et al., 2020; Semagn et al., 2022). This balance reflects the physiological trade-off between growth and defense. Systemic infections like WBD induce hypertrophic growth that acts as a significant metabolic sink, diverting carbon and resources away from pod development. The Pareto-based multiobjective screen and sensitivity analyses identified accessions—such as SCC85, CRICF13, and EET377—that consistently maintained high rankings despite deterministic and random perturbations of index weights. The Pareto-optimal subset, together with the broader rank-stable shortlist, may represent genotypes that better balance disease resilience and reproductive output, thereby maintaining favorable rankings across alternative weighting schemes. The rank-stable accessions identified here should therefore be viewed as prioritized candidates for follow-up evaluation rather than as independently validated resistant selections.

Certain limitations must be acknowledged. The sample size of the phenotyped panel restricts the statistical power to estimate small marker effects and limits the precision of prediction accuracy estimates. This limitation is important because the prediction set contained 102 accessions but more than 8,000 markers per reference framework. Eigenvalue-based summaries indicated that the effective marker dimensionality was approximately 21.3 for Criollo and 16.9 for Matina, highlighting that the analysis was conducted in a strongly structured small-*n*/high-*p* setting. Therefore, the observed prediction accuracies should be interpreted as moderate within this panel rather than as final estimates of deployment accuracy. Additionally, the phenotypic data were derived from a single geographical location over repeated harvests. Thus, the four harvest periods capture temporal variation within one field-evaluation context but do not provide a direct test of macro-environmental genotype-by-environment interaction. Because disease pressure and microclimate can vary across locations, the top-ranked accessions should be considered candidates for further multi-environment validation rather than validated breeding selections.

In conclusion, this study shows that repeated-harvest disease responses in cacao possess a stable temporal structure that can be moderately predicted using genome-wide markers. WBD-related traits, consistent with the pathogen’s systemic infection biology, provided the most robust and FDR-supported prediction targets in this panel. For breeding applications, we recommend prioritizing WBD trajectory intercepts and slopes as candidate genomic-prediction targets and validating the resulting rank-stable accessions in larger multi-environment trials.

## Data Availability

The raw genotypic and phenotypic data analyzed in this study were originally published by Osorio-Guarín et al. (2020) in G3: Genes, Genomes, Genetics (https://doi.org/10.1534/g3.120.401153) and are publicly available through the supplementary materials of that publication. The custom code is openly available at: https://github.com/EJSAHN/cacao-longitudinal-genomic-prediction. The original contributions presented in the study are included in the article/**Supplementary Material**. Further inquiries can be directed to the corresponding author.
