# Supplementary material for: Longitudinal genomic prediction of cacao disease resilience identifies robust witches’ broom disease targets
Source: Front Plant Sci. 2026 Jun 15;17:1837102. doi: 10.3389/fpls.2026.1837102 (PMC13311075; doi:10.3389/fpls.2026.1837102)
Supplement: Supplementary file 2 [file DataSheet1.docx]

**
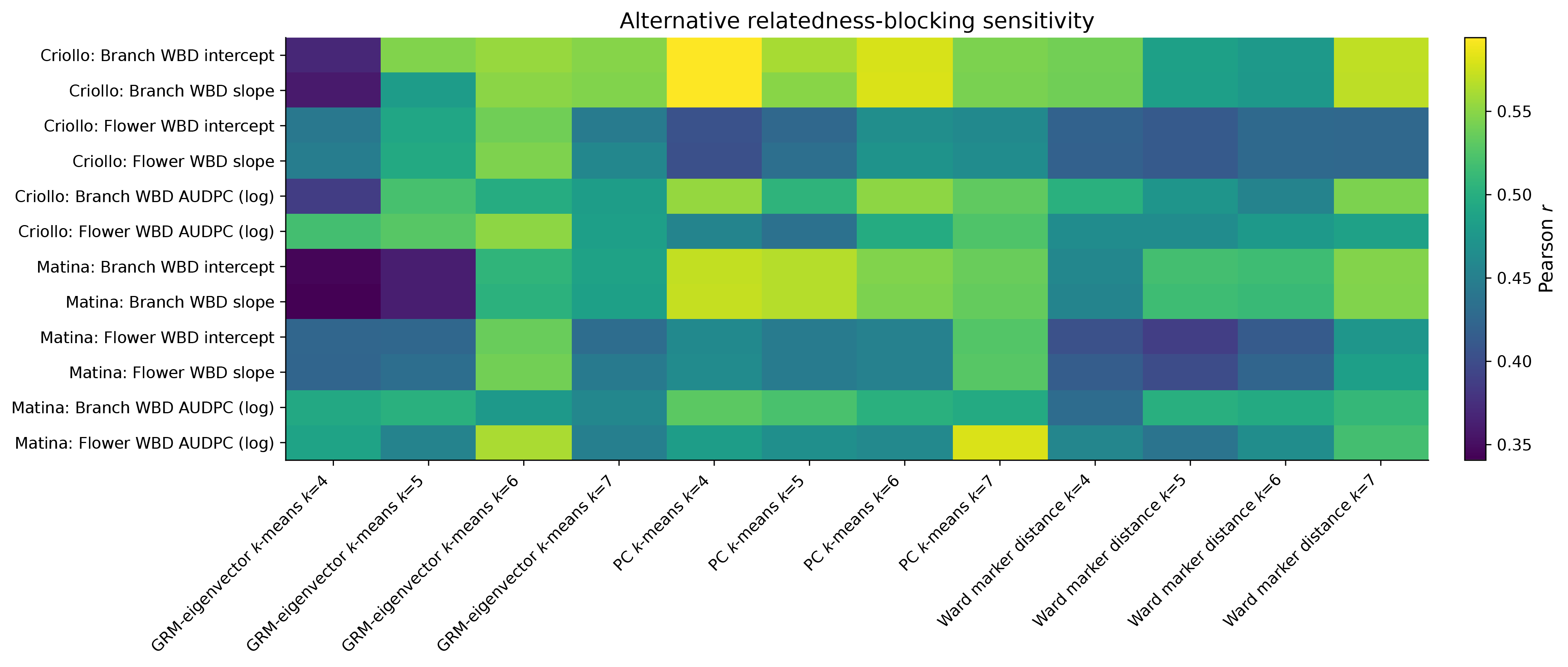
**

**Supplementary Figure S1. Alternative relatedness-blocking sensitivity.**

Blocked-CV Pearson correlations for WBD-derived targets across Ward marker-distance clustering, *k*-means clustering on marker principal components, and *k*-means clustering on relationship-matrix eigenvectors under *k* = 4–7 block definitions.


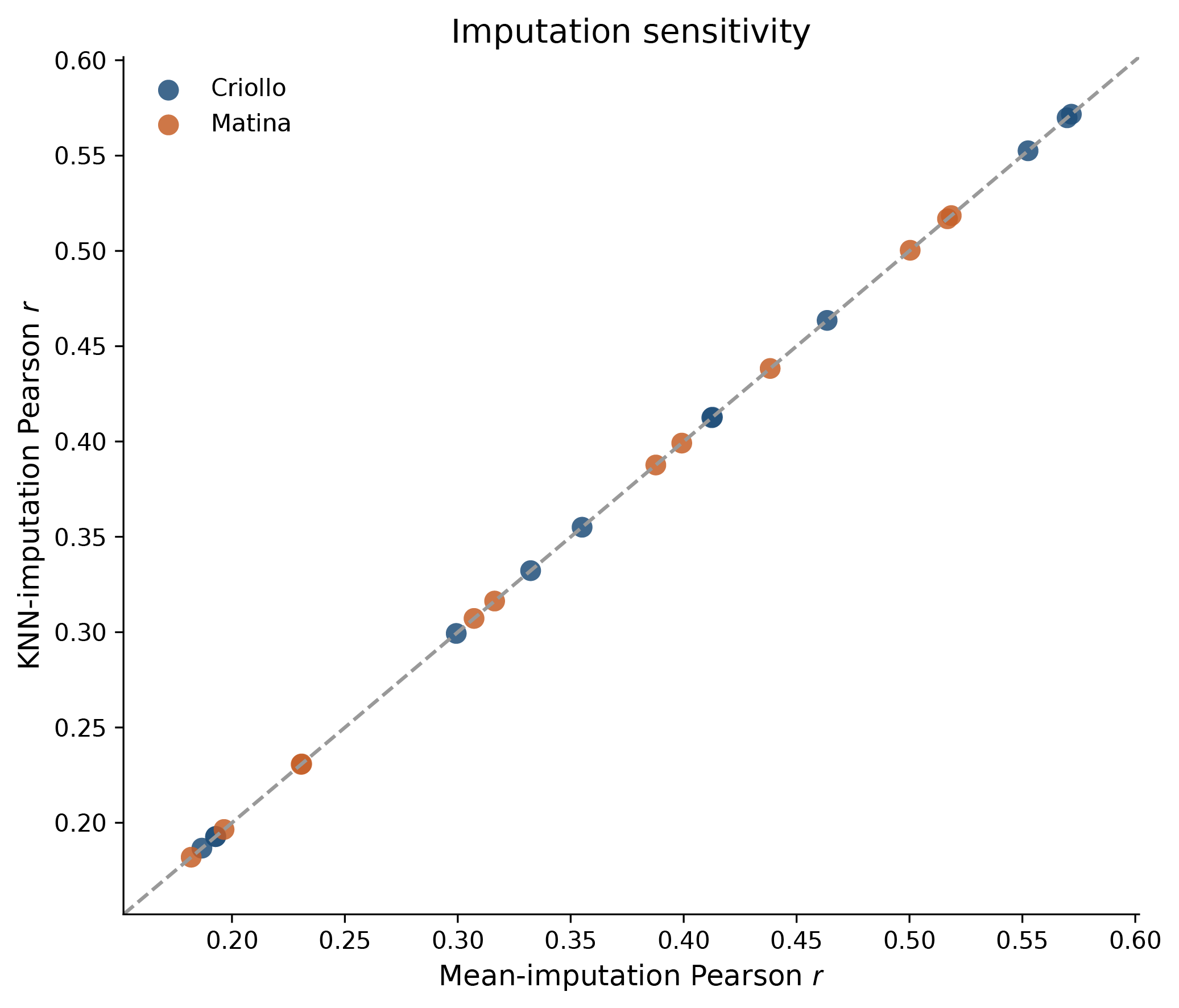


**Supplementary Figure S2. Imputation sensitivity.**

Comparison of relatedness-blocked GBLUP Pearson correlations obtained using marker-mean imputation and KNN imputation. Results were identical or nearly identical because no missing genotypes remained among retained markers in the phenotyped subset after quality filtering.
